# Long noncoding RNA DANCR, working as a competitive endogenous RNA, promotes ROCK1-mediated proliferation and metastasis via decoying of miR-335-5p and miR-1972 in osteosarcoma

**DOI:** 10.1186/s12943-018-0837-6

**Published:** 2018-05-12

**Authors:** Yong Wang, Xiandong Zeng, Ningning Wang, Wei Zhao, Xi Zhang, Songling Teng, Yueyan Zhang, Zhi Lu

**Affiliations:** 1 4th Department of Orthopedic Surgery, Central Hospital affiliated to Shenyang Medical College, No.5, South Seven West Road, Tiexi District, Shenyang, Liaoning 110024 People’s Republic of China; 2 Department of Surgical Oncology, Central Hospital affiliated to Shenyang Medical College, Shenyang, People’s Republic of China; 3 2nd Department of Cardiology, Central Hospital affiliated to Shenyang Medical College, Shenyang, People’s Republic of China; 4 Institute for Cardiovascular Prevention, Ludwig-Maximilians-University, Munich, Germany; 5 1st Department of Hand Surgery, Central Hospital affiliated to Shenyang Medical College, Shenyang, People’s Republic of China; 6 Department of Pathology, Liaoning Cancer Hospital & Institute, Shenyang, People’s Republic of China; 7 Department of Nuclear Medicine, The First Affiliated Hospital of Dalian Medical University, Dalian, 116011 People’s Republic of China

**Keywords:** LncRNA DANCR, miR-335-5p/miR-1972, Osteosarcoma, Proliferation/Metastasis, ceRNA

## Abstract

**Background:**

Accumulating evidences indicate that non-coding RNAs (ncRNAs) including long non-coding RNAs (lncRNAs) and microRNAs (miRNAs) acting as crucial regulators in osteosarcoma (OS). Previously, we reported that Rho associated coiled-coil containing protein kinase 1 (ROCK1), a metastatic-related gene was negatively regulated by microRNA-335-5p (miR-335-5p) and work as an oncogene in osteosarcoma. Whether any long non-coding RNAs participate in the upstream of miR-335-5p/ROCK1 axial remains unclear.

**Methods:**

Expression of differentiation antagonizing non-protein coding RNA (DANCR) and miR-335-5p/miR-1972 in osteosarcoma tissues were determined by a qRT-PCR assay and an ISH assay. Osteosarcoma cells’ proliferation and migration/invasion ability changes were measured by a CCK-8/EDU assay and a transwell assay respectively. ROCK1 expression changes were checked by a qRT-PCR assay and a western blot assay. Targeted binding effects between miR-335-5p/miR-1972 and ROCK1 or DANCR were verified by a dual luciferase reporter assay and a RIP assay. In vivo experiments including a nude formation assay as well as a CT scan were applied to detect tumor growth and metastasis changes in animal level.

**Results:**

In the present study, an elevated DNACR was found in osteosarcoma tissue specimens and in osteosarcoma cell lines, and the elevated DNACR was closely correlated with poor prognosis in clinical patients. Functional experiments illustrated that a depression of DANCR suppressed ROCK1-mediated proliferation and metastasis in osteosarcoma cells. The results of western blot assays and qRT-PCR assays revealed that DANCR regulated ROCK1 via crosstalk with miR-335-5p and miR-1972. Further cellular behavioral experiments demonstrated that DNACR promoted ROCK1-meidated proliferation and metastasis through decoying both miR-335-5p and miR-1972. Finally, the outcomes of in vivo animal models showed that DANCR promoted tumor growth and lung metastasis of osteosarcoma.

**Conclusions:**

LncRNA DANCR work as an oncogene and promoted ROCK1-mediated proliferation and metastasis through acting as a competing endogenous RNA (ceRNA) in osteosarcoma.

**Electronic supplementary material:**

The online version of this article (10.1186/s12943-018-0837-6) contains supplementary material, which is available to authorized users.

## Background

Osteosarcoma (OS) is a prevalent malignant bone tumor whose predictive sites are metaphysis of long bones among adolescents [1]. The character of fast growth and early stage of metastasis takes the main charge for the unfavorable prognosis of osteosarcoma [2]. As a result of its vicious biological behavior, being present of pulmonary metastasis is approximately 20% in the first visit of patients [3]. Consequently, seeking out a crucial metastatic-related molecular and identifying its underlying mechanism in osteosarcoma are compelling needed.

Long non-coding RNAs (lncRNAs), which are a group of transcribed RNA molecules and with length of more than 200 nucleotides, are involved in diverse biological processes in tumor initiation, growth and metastasis through epigenetic, transcriptional and post transcriptional mechanisms [4–6]. lncRNAs present multiple functions including post-transcriptional regulation, chromatin modification in various malignant tumors such as lung adenocarcinoma, esophageal squamous cell carcinoma, renal cell carcinoma, hepatocellular carcinoma and osteosarcoma [7–11]. Among the working mechanisms of lncRNAs, competing endogenous RNA (ceRNA) theory which was first proposed by Leonardo Salmena received high recognition in noncoding RNA area worldwide [12]. CeRNA theory hypothesizes that RNA transcriptions includes lncRNAs communication through a new “language” mediated by microRNA response elements (MREs).

Previous studies by our group indicated that miR-335 functioned as a tumor suppressor by way of directly targeting to its downstream gene - Rho associated coiled-coil containing protein kinase 1 (ROCK1) [13, 14]. Here, we wondered whether lncRNA could interact with miR-335 via the mechanism of ceRNA. Online prediction driving differentiation antagonizing non-protein coding RNA (DANCR) - a member of lncRNAs - to arouse our attention for its similar MREs which might be provided for miR-335-5p as ROCK1 did. Even further, we found that DANCR and ROCK1 shared two MREs for two miRNAs, miR-335-5p and miR-1972, respectively. In the present study, we focused on the ceRNA network which is comprising of DANCR, miR-335-5p/miR-1972 and ROCK1, as well as explored the potential effect of DANCR to ROCK1 - mediated proliferation and migration/invasion in osteosarcoma.

## Methods

### Patients and tissue samples

Ninety-five cases of osteosarcoma tissues and matched para-tumor tissues used in this study were collected under the permissions of patients during tumorectomy in Liaoning Cancer Hospital & Institute from February 2013 to September 2017. None of the patients had received chemotherapy before surgery and all the 95 cases were diagnosed based on a definite pathological diagnosis and the clinical stages of these patients were determined according to the tumor.node.metastasis (TNM) classification of the International Union Against Cancer (UICC). Written informed consent was obtained from all participants. The Institute Research Medical Ethics Committee of Central Hospital affiliated to Shenyang Medical College and Liaoning Cancer Hospital & Institute granted approval for this study.

### Cell culture

Human osteosarcoma cell lines MG-63, U2OS, MNNG/HOS, 143B and human osteoblast cell line hFOB 1.19 were purchased from the Chinese Academy of Sciences Cell Bank (Shanghai, China) and maintained in DMEM/F12 (Gibco, El Paso, TX, USA), DMEM (Gibco), DMEM, MEM, (Gibco) and DMEM, respectively. All mediums were supplemented with 10% (*v*/v) fetal bovine serum (FBS, Sigma, St. Louis, MO, USA), 100 IU/ml penicillin (Baomanbio, China) and 100 mg/ml streptomycin (Baomanbio, China). All osteosarcoma cell lines were cultured at 37 °C in a humidified atmosphere containing 5% CO_2_ while human osteoblast cell line hFOB 1.19 was cultured at 34 °C with 5% CO_2_.

### Plasmids and cell transfection

MiR-335-5p mimics, miR-1972 mimics, NC mimic, miR-335-5p inhibitor, miR-1972 inhibitor and NC inhibitor were purchased from Riobio (Ribobio, Guangzhou, China). DANCR overexpression plasmids pcDNA-DANCR, wt-DNACR-335, wt-DANCR-1972, mut-DANCR-335 and mut-DANCR-1972 as well as DANCR and ROCK1 knockdown plasmids DANCR shRNA and ROCK1 shRNA with a corresponding negative control shRNA (NC shRNA) were chemically synthesized by GenePharma (GenePharma, Shanghai, China). The sequence of DANCR shRNA and ROCK1 shRNA were listed in Additional file 1: Table S1. Quantitative real-time PCR analysis was applied to detect DANCR and ROCK1 expression level to value the silenced efficiencies. The results were shown in Additional file 2: Figure S2A, DANCR shRNA - 01 was selected as the optimum shRNA in the subsequent experiments due to the highest suppressive effect. ROCK1 shRNA-01 was selected by the same means as presented in Additional file 2: Figure S2B and was employed in following ROCK1 RNAi experiment. The aforementioned plasmids were transfected into MNNG/HOS and 143B cells by Lipofectamine 2000 (Invitrogen, Carlsbad, CA, USA) according to the manufacturer’s protocol. For a stable cell transfection, cells were selected by the culture medium containing 0.4 mg/ml Geneticin (G418; Invitrogen). After 6 weeks, G418-resistant cell clones were established.

### Reverse transcription and quantitative real-time PCR

The procedure was carried out as previously described [15]. Total RNAs were extracted using Trizol reagent (Invitrogen, Carlsbad, CA, USA). cDNAs were synthesis by PrimeScriptTM RT reagent kit (Takara, Dalian, China). qPCR was performed with the SYBR Premix Ex Taq II kit (TaKaRa) and the Applied Biosystems 7500 Fluorescent Quantitative PCR system (Applied Biosystems Life Technologies, USA). GAPDH and U6 were used to normalize the expression levels of the DANCR/ROCK1 and miR-335-5p/miR-1972, respectively. Primers sequences were listed in Additional file 1: Table S1.

### Western blot analysis

Total proteins were isolated using radio immunoprecipitation assay (RIPA) lysis buffer (Sigma, St. Louis, MO, USA) and qualified by BCA detecting kit (Keygen, Nanjing, China) flollowing to the manufacture’s protocol. Proteins samples were subjected to 10% SDS-PAGE and transferred onto a PVDF membrane, then incubated with ROCK1 (Abcam, Cambridge, MA, UK; dilution rates of 1:2000) and GAPDH antibodies (Abcam, dilution rates of 1:500) at 4 °C overnight, respectively. The next day, the membranes were incubated with secondary antibodies (Abcam, dilution rates of 1:2000) at room temperature for 1 h. Protein bands were detected on X-ray film via enhanced chemiluminescence detection system.

### Cell proliferation assays

Osteosarcoma cells’ changes of proliferative capacity were determined by 5-Ethynyl-20-Deoxyuridine (EDU) incorporation assay and cell counting Kit-8 (CCK-8) assay. For EDU incorporation assay, the procedure was carried out according to the manufacturer’s instructions with EDU detection kits (Keygen, Nanjing, China). CCK-8 assay was executed as previously reported [16]. In brief, osteosarcoma cells were seeded in 96-well plates (2 × 10^3^) supplemented with complete growth medium and followed by different transfection 24 h later. At days 1, 2, 3, 4 and 5 after transfection, 10 μl CCK-8 solution was added into each well and incubated for 2 h. The absorbance was measured at an optical density of 450 nm using Microplate reader (Bio-Rad, CA, USA). Experiments were repeated in triplicate.

### Transwell assays

The procedure was carried out as previously described [5]. In short, MNNG/HOS and 143B cells were seeded on uncoated (for migration assays) and Matrigel-coated (for invasion assays) upper chambers (BD Bioscience, New Jersey, USA) respectively. Culture medium non-containing and containing 10% FBS were supplemented into the upper and lower wells respectively and incubated for further 24 h. Followed by wiping of non-migrated or non-invaded cells. Then the filters were fixed in 90% ethanol and followed by crystal violet staining. Five random fields were counted per chamber by using an inverted microscope (Olympus, Tokyo, Japan).

### In situ hybridization assay and immunohistochemistry analysis

In situ hybridization assay was performed on fresh osteosarcoma tissue slices. In brief, slices were washed with1× phosphate buffered saline (PBS)containing 0.5% Triton X-100, afterwards be incubated with appropriate amount of anti-DANCR, anti-miR-335-5p and anti-miR-1972 oligodeoxy-nucleotide probes (RiboBio, Guangzhou, China) with hybridization solution containing 1% blocking solution in humid chamber at 37 °C overnight. The next day, slices were then washed three times for 5 min each at 42 °C with 0.1% Tween-20 in 4× sodium citrate buffer (SSC), once for 5 min in 2× SSC and once for 5 min in 1× SSC in dark. Following, after be rinsed with 1 × PBS for 5 min triply at room temperature and be re-stained by DAPI (Cell Signaling Technologies, Danvers, USA), fluorescent microscope (Leica, Wetzlar, Germany) was applied to observe and photograph the slices. Images were analyzed using Image - Pro Plus 6.0 software (Media Cybernetics, Rockville, USA).

The procedure of immunohistochemistry was performed as previously described [16].

### Dual luciferase reporter assay

The procedure was carried out as previously described [10]. Wild and mutant reporter plasmids of DANCR (wt-DANCR-335-luc, mut-DANCR-335-luc, wt-DANCR-1972-luc and mut-DANCR-1972-luc) and ROCK1 (wt-ROCK1–335-luc, mut-ROCK1–335-luc, wt-ROCK1–1972-luc and mut-ROCK1–1972-luc) which containing a wild or mutant miR-335-5p or miR-1972 binding sites were individually synthesized by GenePharma (GenePharma, Shanghai, China). When HEK293 cells achieved to 70% confluence, the synthesized reporter plasmids were co-transfected with miR-335-5p mimics/miR-1972 mimics or mimic control respectively by Lipofectamine 2000 (Invitrogen). Forty-eight hours later, the changes of the fluorescence in each group were detected using Dual-Luciferase Reporter Assay System (Promega, Madison, WI, USA) according to the manufacturer’s protocol.

### RNA immunoprecipitation (RIP) assay

The procedure was carried out as previously described using Magna RNA-binding protein immunoprecipitation kit (Millipore, Billerica, MA, USA) [17]. In brief, whole-cell lysate was incubated with RIP buffer containing magnetic beads conjugated with human anti-Ago2 antibody, or normal mouse IgG as negative control. Samples were incubated with proteinase K and then immunoprecipitated RNA was isolated. The RNA concentration was measured by spectrophotometer (Thermo Scientific, Waltham, MA, USA) and the RNA quality was assessed with bio-analyzer (Agilent, Santa Clara, CA, USA). Furthermore, purified RNAs were extracted and analyzed by quantitative real-time PCR to demonstrate the presence of binding targets.

### In vivo nude mouse models

Female nude mice (4–5 weeks old) were purchased from Animal Care and Use Committee of Dalian Medical University Ltd., and kept under sterile specific-pathogen-free (SPF) conditions. 1 × 10^6^ 143B cells (mixed with Matrigel, BD Bioscience, 1:1) with stable overexpression of DANCR (DANCR puro) and with corresponding blank vector (pMSCV puro) were injected subcutaneously or intravenously for the determination of tumor growth and metastasis. Tumor formation and metastasis were monitored by Fluorescent Imager (IVIS Spectrum, Caliper Life Sciences, Hopkinton, MA, once per week for 4 weeks) and Computed Tomography (CT, Siemens, München, Germany, at week four) scan, respectively. The subcutaneous formatted tumor nodes and the lungs from metastatic group were harvested for further detection. This study was carried out in accordance with the Guide for the Care and Use of Laboratory Animals of the National Institutes of Health and was granted by the Institute Research Medical Ethics Committee of Central Hospital affiliated to Shenyang Medical College.

### Statistical analysis

The significance of differences between groups was assessed by Student’s t-test, one-way ANOVAs or χ^2^ test, as appropriate. Survival curves were estimated by the Kaplane-Meier method. The log-rank test was used to determine the statistical differences between survival curves. All statistical analyses were performed using GraphPad Prism V5.0 (GraphPad Software, Inc., La Jolla, CA, USA) and SPSS 19.0 (IBM, SPSS, Chicago, IL, USA). A two-tailed *P* < 0.05 was considered as statistically significant while *P* < 0.01 was very significant.

## Results

### DANCR was elevated in osteosarcoma tissues and cell lines and was correlated with poor prognosis in osteosarcoma patients

**Fig. 1:**
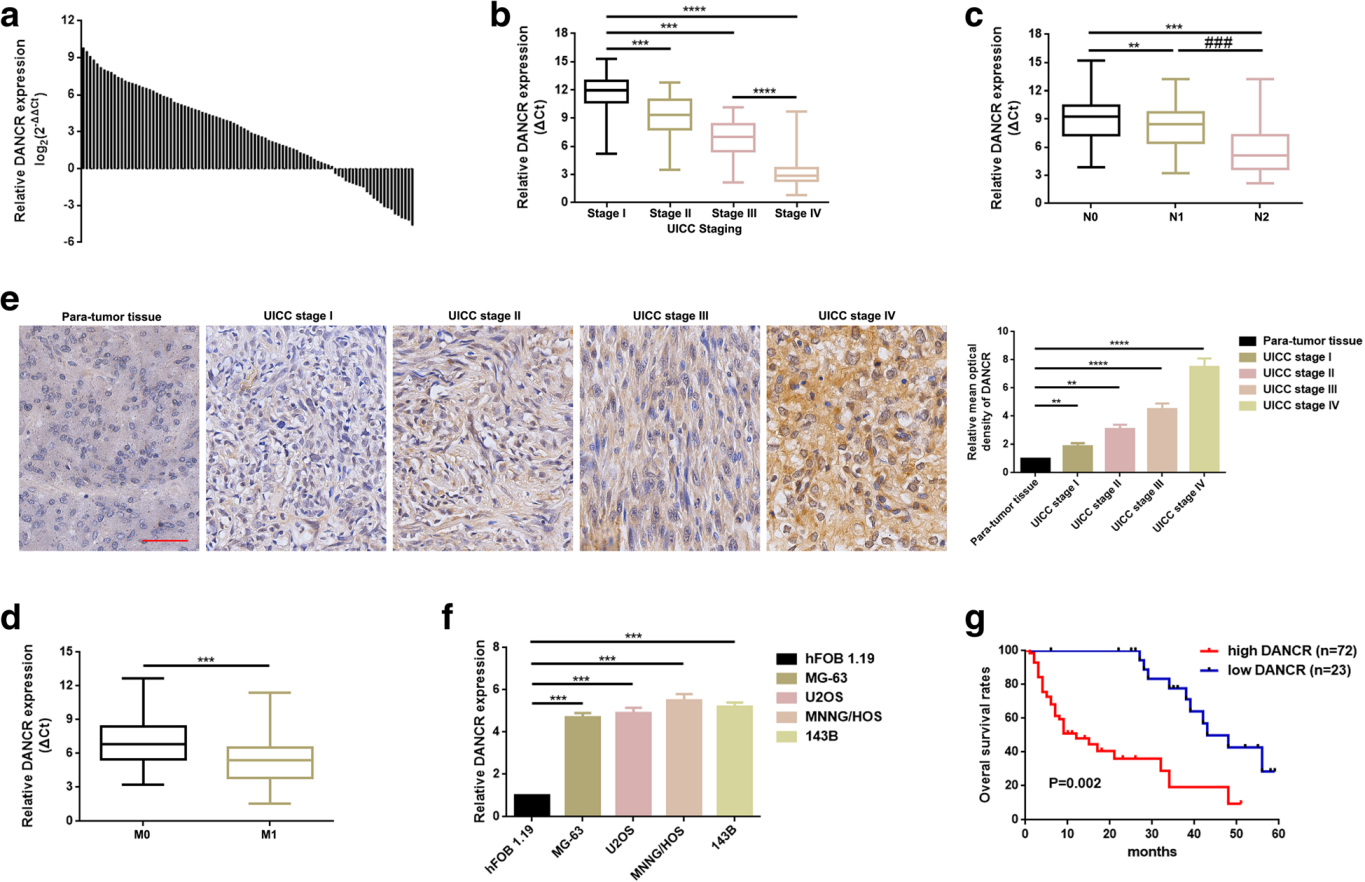
**a** DANCR expression in 95 cases of osteosarcoma tissues and paired para-tumor tissues were determined by using of qRT-PCR, data were shown as log_2_ (2^-△△Ct^). **b**-**d** DANCR expression was significantly elevated in patients with advanced stage (**b**) (***, *P* < 0.001,****, *p* < 0.0001), lymph node metastasis (**c**) (**, *P* < 0.01,***, *P* < 0.001,) and distant metastasis (**d**) (**, *P* < 0.01,***, *P* < 0.001, ###, *P* < 0.001, respectively) with data be shown as △Ct. **e** DANCR expression was gradually elevated with advanced staging as detected by In situ hybridization analysis, **, *P* < 0.01, ***, *P* < 0.001, as comparing with para-tumor tissue group, respectively. **f** Elevated DANCR was presented in osteosarcoma cell lines contrasted to hFOB 1.19 group, data was normalized to hFOB 1.19 group, ***, *P* < 0.001. **g** Kaplan-Meier analyses indicated that overall survival (OS) in the patients with high DANCR was significantly shorter than that in the patients with low DANCR, *P* = 0.02. Data were shown as mean ± SD from three independent experiments

The expression of DANCR in collected 95 cases of osteosarcoma tissues and matched para-tumor tissues were determined by applying of quantitative real-time PCR (qRT-PCR) as normalizing to GAPDH. As the results presented in Fig. 1a, DANCR was elevated in most osteosarcoma tissues (72/95, 75.79%) when compared with para-tumor tissues. Additionally, the potential correlation between expression level of DANCR and clinicopathological features were uncovered. As the outcomes displayed in Fig. 1b-d, the expression of DNACR was notably higher in patients with advanced stage, lymph node metastasis and distant metastasis. Meanwhile, further analysis by Pearson Chi-Square test or Fisher’s Exact Test indicated higher expression of DANCR was correlated with bigger tumor size (≥ 5 cm), advanced staging (IIB/III) and distant metastasis (Table 1). Moreover, In situ hybridization (ISH) analysis was applied to evaluate the expression of DANCR in tissue level. As shown in Fig. 1e, DANCR was gradually strongly stained with staging in osteosarcoma tissues than that in para-tumor tissues. Also, DANCR was intensively stained in osteosarcoma cells’ cytoplasm. Even further, the expression level of DANCR in 4 osteosarcoma cell lines MG-63, U2OS, MNNG/HOS, 143B and a normal human osteoblastic cell line hFOB 1.19 were also measured by qRT-PCR. As was illustrated in Fig. 1f, the expression of DANCR was significantly elevated in osteosarcoma cell lines MG-63, U2OS, MNNG/HOS, 143B compared to that in hFOB 1.19. Finally, Kaplan-Meier analysis and log-rank test were used to evaluate the correlation between the elevated DANCR and the final survival time of osteosarcoma patients. The results in Fig. 1g suggested that high DANCR expression was inversely correlated with osteosarcoma patients’ overall survival.

**Table 1:** Association of DANCR expression with clinicopathological features of osteosarcoma

Features	No. of cases	DANCR		*p* value†
		High	Low	
Age at diagnosis				0.481
<18	52	40	12	
≥18	43	32	11	
Gender				0.472
Female	51	38	13	
Male	44	34	10	
Histological subtype				0.674
Osteoblastic	9	6	3	
Chondroblastic	18	12	6	
Fibroblastic	38	30	8	
Mixed	30	24	6	
Clinical stage				0.005
I + IIA	42	26	16	
IIB/III	53	46	7	
Distant metastasis				0.002
Absent	37	22	15	
Present	58	50	8	
Tumor size (cm)				0.014
<5	39	23	16	
≥ 5	56	49	7	
Anatomic location				0.431
Tibia/femur	49	38	11	
Elsewhere	46	34	12	

†*P*-value obtained from Pearson Chi-Square test or Fisher’s Exact Test

### DANCR promoted proliferation and migration/invasion in MNNG/HOS and 143B cells

**Fig. 2:**
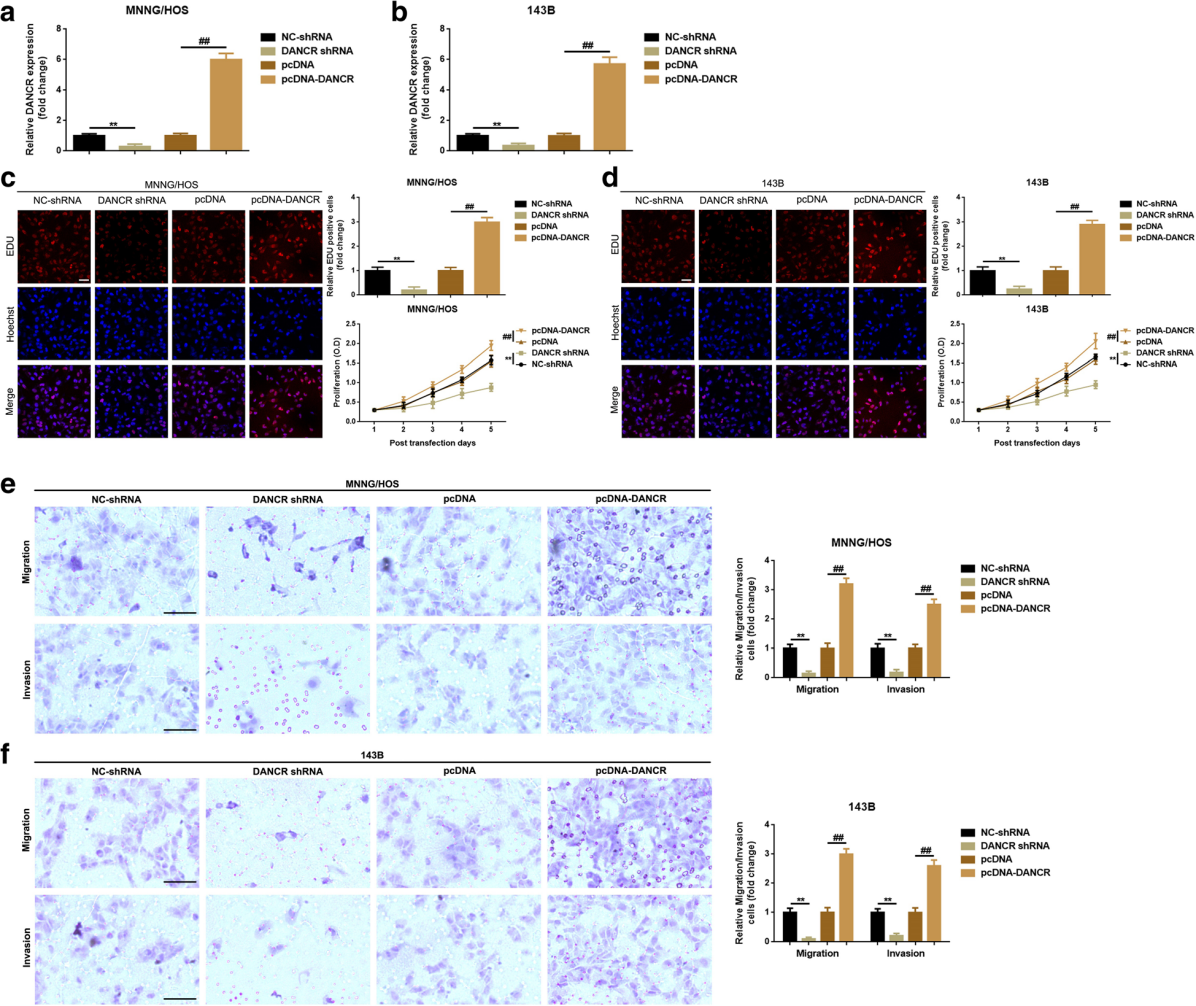
**a**-**b** Over-expression and knockdown of DANCR in MNNG/HOS (**a**) and 143B (**b**) cells were confirmed by qRT-PCR. **c**-**d** EDU assay and CCK-8 assay were performed to determine the cell proliferation changes after DANCR up- and down-regulation in MNNG/HOS (**c**) and 143B (**d**) cells. **e**-**f** Transwell assay was applied to evaluate the migration/invasion ability changed after DANCR regulation in MNNG/HOS (**e**) and 143B (**f**) cells. **, *P* < 0.01 and ##, *P* < 0.01, normalized to NC-shRNA and pcDNA group, respectively. Data were shown as mean ± SD from three independent experiments

Since DANCR was highly expressed in osteosarcoma and was correlated with tumor size and distant metastasis in clinical cases, we wondered about the function DANCR might act on osteosarcoma cells’ proliferation and metastasis. Up-regulation and down-regulation of DANCR by transfection of DANCR-shRNA and pcDNA-DANCR respectively were confirmed by qRT-PCR (Fig. 2a-b). Then, 5-ethynyl-2′-deoxyuridine (EdU) incorporation assays and CCK-8 assays were applied to evaluate the effects of DANCR on cell proliferation in osteosarcoma cells MNNG/HOS and 143B. As was presented in Fig. 2c-d, DANCR confidently enhanced the proliferative capacity in MNNG/HOS and 143B cells, and vice versa. In addition, the migration/invasion ability changes in MNNG/HOS and 143B cells were determined by transwell assay. As was shown in Fig. 2e-f, depression and elevation of DANCR also positively regulated osteosarcoma cells’ migration/invasion.

### DANCR promoted proliferation and migration/invasion via up-regulation of ROCK1 in MNNG/HOS and 143B cells

**Fig. 3:**
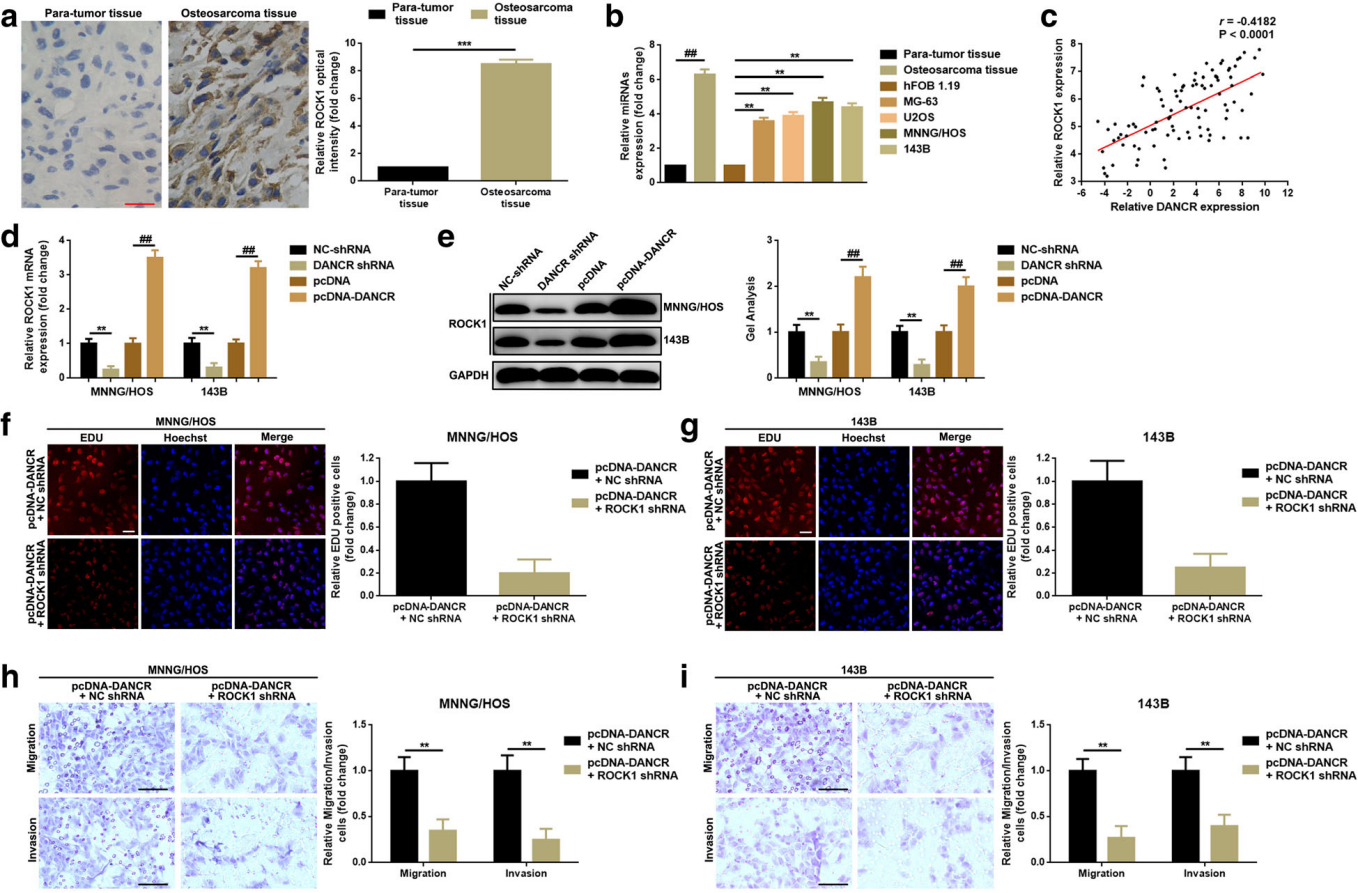
**a** The elevated expression of ROCK1 in tissue level were displayed by an IHC detection, ***, *P* < 0.001, normalized to para-tumor tissue group. **b** ROCK1 was up-regulated in osteosarcoma tissue and cell lines as determined by a qRT-PCR, **, *P* < 0.01 and ##, *P* < 0.01, normalized to para-tumor tissue group and hFOB 1.19 group, respectively. **c** A positive correlations between DNACR and ROCK1 was confirmed by a Pearson correlation analysis, *P* < 0.0001. **d**-**e** The expression of ROCK1 after DANCR regulation were measured by using of qRT-PCR (**d**) and western blot (**e**) in MNNG/HOS and 143B cells, separately. **f**-**g** RNAi technique was used to define the function ROCK1 might work on DANCR induced proliferation. Silencing ROCK1 by transfection of ROCK1 shRNA attenuated DANCR-induced facilitation of proliferation in MNNG/HOS (**f**) and 143B (**g**) cells, as determined by an EDU assay. **h**-**i** A transwell assay was executed to verify the role of ROCK1 in DANCR-induced metastasis in MNNG/HOS (**h**) and 143B (**i**) cells. **, *P* < 0.01, normalized to pcDNA-DANCR + NC shRNA group. Data were shown as mean ± SD from three independent experiments

Increasing evidences demonstrated that lncRNAs regulated various downstream genes to exert multiple functions. According to our and other previous researches, ROCK1 was closely related to osteosarcoma cells’ proliferation and migration/invasion [5, 18]. Here, we attempted to figure out whether the regulation effect of DANCR on osteosarcoma cells’ proliferation and migration/invasion was achieved through ROCK1. We firstly demonstrated that ROCK1 was elevated in osteosarcoma tissue specimens and in osteosarcoma cell lines (Fig. 3a-b). Also, we displayed the positive correlation between DANCR and ROCK1 (Fig. 3c). We then confirmed that up- and down-regulation of DANCR could positively affected ROCK1 expression both in mRNA and in protein level (Fig. 3d-e). Furthermore, we determined the role which ROCK1 might play in DANCR-related proliferation and migration/invasion. As the results presented in Fig. 3f-g, knockdown of ROCK1 (co-transfection of pcDNA-DANCR and ROCK1 shRNA) attenuated the proliferating-assisted effect DANCR in MNNG/HOS and 143B cells. Meanwhile, transwell assay elucidate a similar tendency of ROCK1 performing on migration/invasion as it did on proliferation (Fig. 3h-i).

### DANCR up-regulated ROCK1 via crosstalk with miR-335-5p and miR-1972

**Fig. 4:**
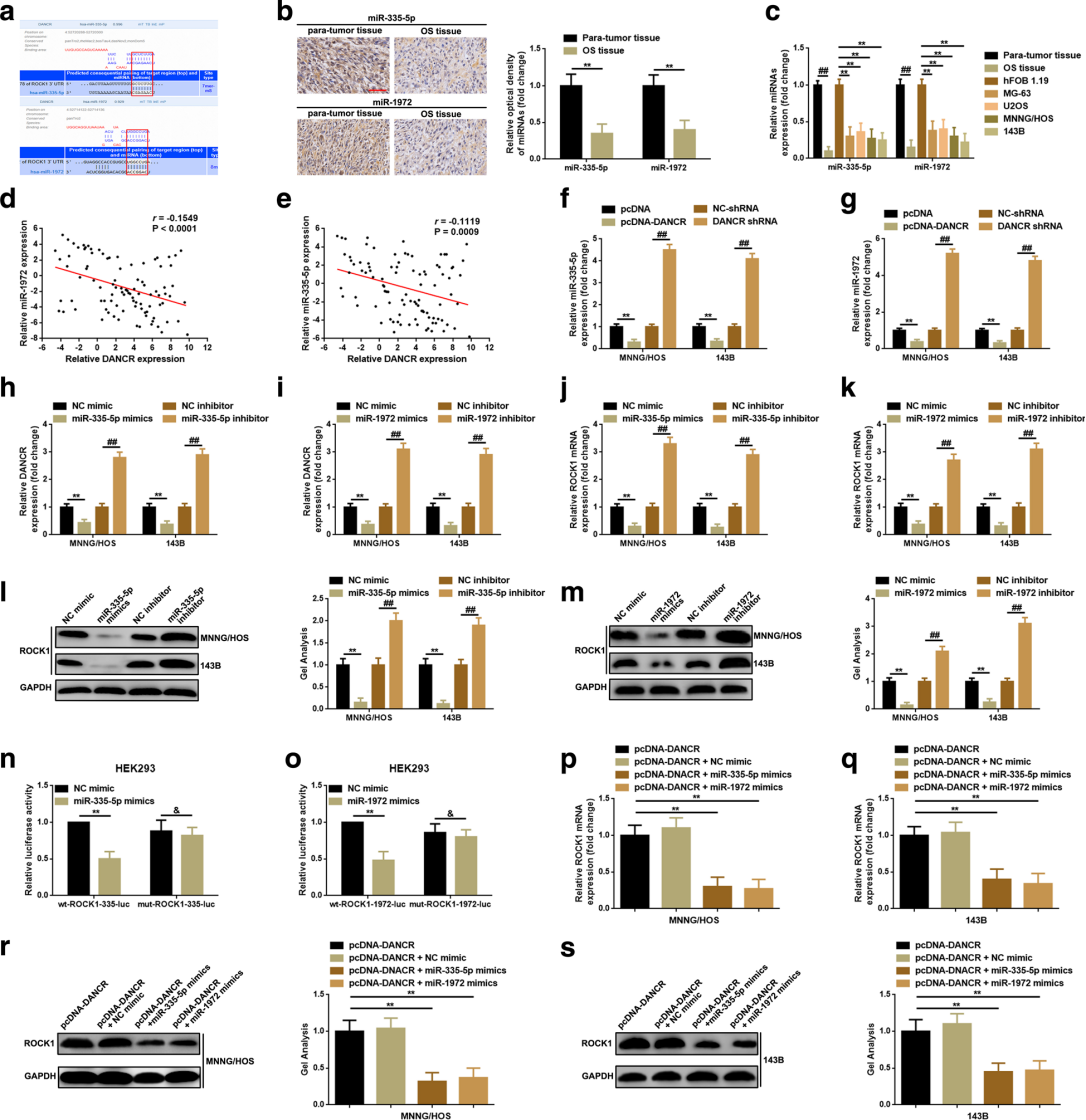
**a** DANCR and ROCK1 shared two similar MREs for miR-335-5p and miR-1972 as predicted by DIANA-LncBase (http://carolina.imis.athena-innovation.gr) and TargetScan (http://www.targetscan.org/vert_71), individually. **b** miR-335-5p and miR-1972 in tissue level were detected by using ISH, **, *P* < 0.01, normalized to para-tumor tissue group. **c** The expression of miR-335-5p and miR-1972 in osteosarcoma tissue and cell lines were assessed by qRT-PCR, ##, *P* < 0.01 and **, *P* < 0.01 as normalized to para-tumor tissue group and hFOB 1.19 group, separately. **d**-**e** Correlations between DNACR and miR-335-5p (**d**) as well as DANCR and miR-1972 (**e**) were qualified by a Pearson correlation analysis, *P* < 0.0001 for miR-335-5p and *P* = 0.0009 for miR-1972, respectively. **f**-**g** A qRT-PCR assay verified that increased or reduced DNACR negatively regulated miR-335-5p (**f**) and miR-1972 (**g**) expression, **, *P* < 0.01 and ##, *P* < 0.01 as normalized to pcDNA group and NC shRNA group. **h**-**i** An elevation and a depression of miR-335-5p (**h**) and miR-1972 (**i**) also inversely regulated DANCR expression, separately. Determined by qRT-PCR, **, *P* < 0.01 and ##, *P* < 0.01 as normalized to mimic control group and inhibitor control group. **j**-**m** Up-regulation or down-regulation of miR-335-5p (**j** and **l**) and miR-1972 (**k** and **m**) functioned on ROCK1 expression inversely as measured by qRT-PCR and western blot. **, *P* < 0.01 and ##, *P* < 0.01 as normalized to mimic control group and inhibitor control group. **p**-**s** Elevation of miR-335-5p and miR-1972 significantly attenuated the facilitative effect of DANCR on ROCK1 expression as determined by qRT-PCR (**p** and **q**) and western blot (**r** and **s**) in MNNG/HOS and 143B cells, respectively. **, *P* < 0.01, normalized to pcDNA-DANCR group. Data were shown as mean ± SD from three independent experiments

Lately, researches on the competitive endogenous RNA (ceRNA) theory between lncRNAs and miRNAs are the hotspots in the field of noncoding RNAs. Therefore, we specuated whether DANCR might crosstalk with any potential miRNAs in dependence of the same mechanism in osteosarcoma. Firstly, an online lncRNA prediction software (DIANA-LncBase) was applied to predict the potential miRNAs that might interact with DANCR. Among the top 18 miRNAs of which the predictive binding scores were higher than 0.9 (Additional file 3: Figure S1), miR-335-5p and miR-1972 stroke our sight because DANCR and ROCK1 shared the similar miRNA response elements (MREs) for miR-335-5p and miR-1972 (Fig. 4a, predicted by DIANA-LncBase and TargetScan). Secondly, the expression of miR-335-5p and miR-1972 in osteosarcoma tissues and cell lines were determined by ISH and qRT-PCR. As was shown in Fig. 4b-c, miR-335-5p and miR-1972 were lower expressed in osteosarcoma tissue and cell lines, individually. And this lower expression level of miR-335-5p and miR-1972 indirectly provided the possibility that DANCR might co-work with them in the progression of osteosarcoma. Thirdly, inverse correlations between DANCR and miR-335-5p as well as DANCR and miR-1972 were presented by Pearson correlation analysis in the collected 95 cases of osteosarcoma tissues (Fig. 4d-e). We then verified that increase or decrease DANCR negatively regulated miR-335-5p and miR-1972 (Fig. 4f-g). Conversely, up-regulated and down-regulated miR-335-5p and miR-1972 also inversely affected in DANCR expression (Fig. 4h-i). These outcomes indicated that DNACR can “crosstalk” with miR-335-5p and miR-1972 in a reciprocal suppression manner. MiRNAs are well known to regulate targeted downstream genes by directly binding to the 3′ untranslated regions (3′UTRs) [19–21]. So, we further proved that miR-335-5p and miR-1972 could negatively regulate ROCK1 mRNA expression and that ROCK1 was a direct target of miR-335-5p and miR-1972 (Fig. 4j-m). Finally, miR-335-5p and miR-1972 mimics were used to detect the effect they might execute on DANCR-induced ROCK1 elevation. As we expected, opposite to NC mimic, miR-335-5p mimics and miR-1972 mimics attenuated DANCR induced ROCK1 elevation (Fig. 4n-o). Together, the evidence in this section demonstrated that DANCR promoted ROCK1 expression via “crosstalk” with miR-335-5p and miR-1972.

### DANCR decoyed miR-335-5p and miR-1972 to facilitate ROCK1-mediated proliferation and migration/invasion

**Fig. 5:**
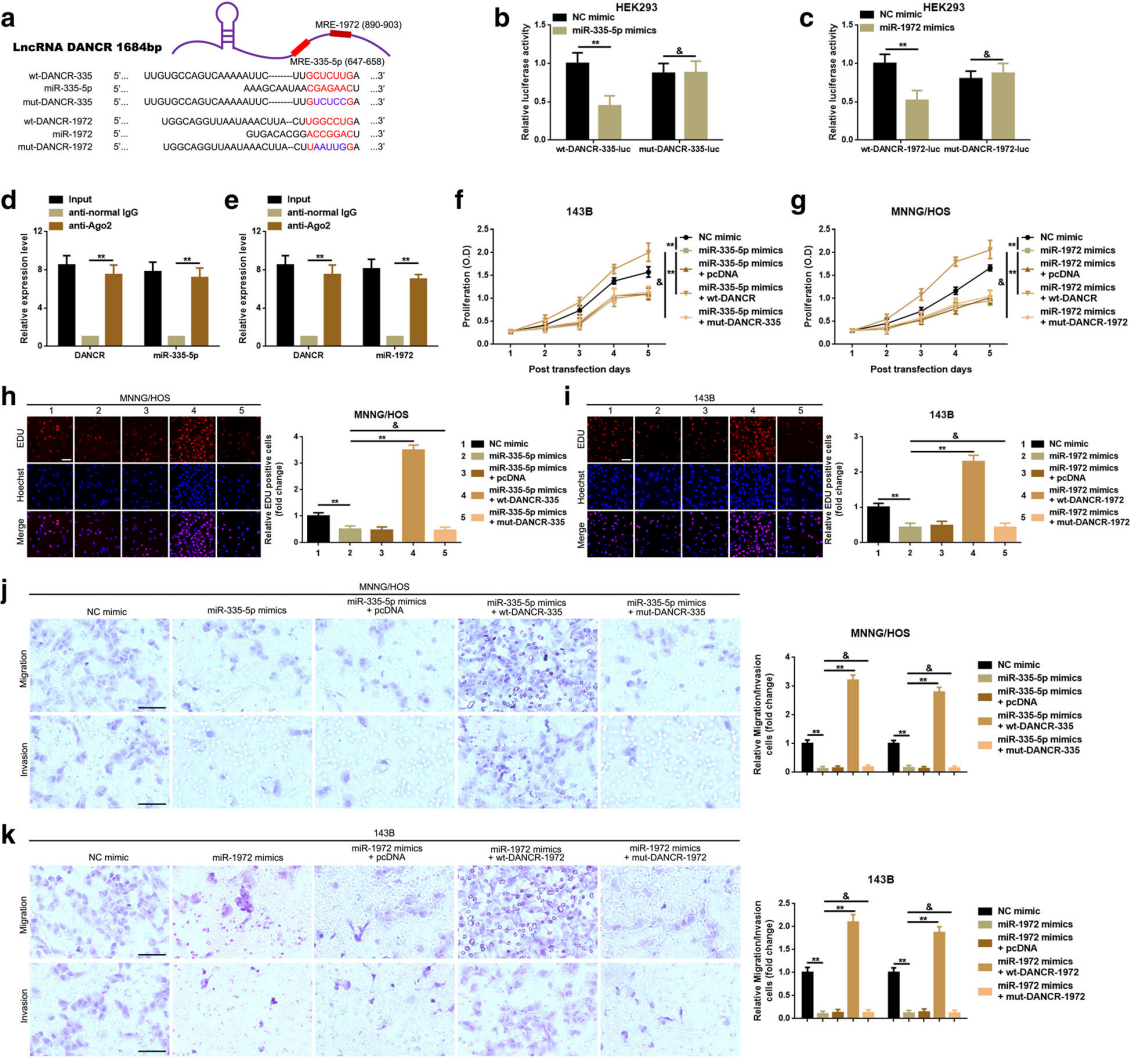
**a** Sequence alignment of DANCR with potential wild type and mutant type of miR-335-5p (wt-DANCR-335 and mut-DANCR-335) and miR-1972 (wt-DANCR-1972 and mut-DANCR-1972) targeting sites. **b**-**c** Luciferase reporter assay was applied to verify the targeted binding effect between DANCR 3′UTR and miR-335-5p or miR-1972, **, *P* < 0.01, as compared and normalize to mimic control group, respectively. **d**-**e** RIP binding assay was performed using input from cell lysate, normal mouse IgG or anti-Ago2. Relative expression levels of DANCR and miR-335-5p or miR-1972 were determined by qRT-PCR. **P < 0.01 vs anti-normal IgG group. **f**-**i** A re-executed CCK-8 and EDU assay presented that promotion of miR-335-5p or miR-1972 (transfection of miR-335-5p mimics or miR-1972 mimics) led to significantly suppression of proliferation, while the suppressive effect was reversed by wt-DANCR but not be done by mut-DANCR-335 or mut-DANCR-1972. **j**-**k** Re-executed transwell assay also demonstrated that it was wt-DANCR that can attenuate miR-335-5p- or miR-1972-induced inhibition of metastasis, when the theoretical binding sites DANCR might provide for miR-335-5p or miR-1972 were mutated, the reversing effect vanished. For F-K, data were normalized to NC mimic group, and **P < 0.01, & *P* > 0.05 as comparing with miR-335-5p mimics group or miR-1972 mimics group, respectively. Data were shown as mean ± SD from three independent experiments

In the above sections, we verified that DANCR could “crosstalk” with miR-335-5p and miR-1972 and promoted ROCK1-mediated proliferation and migration/invasion. In this section, we tried to elucidate whether the ceRNA mechanism do exist among DANCR, miR-335-5p/miR-1972 and ROCK1. We had proved that DANCR affected miR-335-5p and miR-1972 with a reciprocal suppression manner in the previous section, we here applied a luciferase assay to detect the specific binding sites between DNACR and miR-335-5p/miR-1972. As the results showed in Fig. 5b-c, compare to NC mimic, cotransfection of miR-335-5p or miR-1972 mimics and wt-DANCR led to a prominent decrease of fluorescence. When the theoretical binding sites DANCR might provide for miR-335-5p or miR-1972 were mutated (cotranfection of miR-335-5p mimics and wt-DANCR-335 or cotransfection of miR-1972 mimics and wt-DANCR-1972), the fluorescence was restrengthened. Further, as the outcomes of a RIP binding assay presented, the level of DANCR and miR-335-5p/miR-1972 was higher in anti-Ago2 group than that in anti-normal IgG group which indicating that DANCR and miR-335-5p/miR-1972 were in the same RNA induced silencing complex (RISC) (Fig. 5d-e). Combined with the findings above, we believed that DANCR decoyed miR-335-5p and miR-1972 to promote their common-target gene - ROCK1. Furthermore, an antisense experiment was executed to final support the hypothesis that DANCR improved ROCK1-mediated proliferation and migration/invasion via directly decoying of miR-335-5p and miR-1972 in osteosarcoma cells.

### Elevation of DANCR promoted tumorigenesis and lung metastasis of osteosarcoma in vivo

**Fig. 6:**
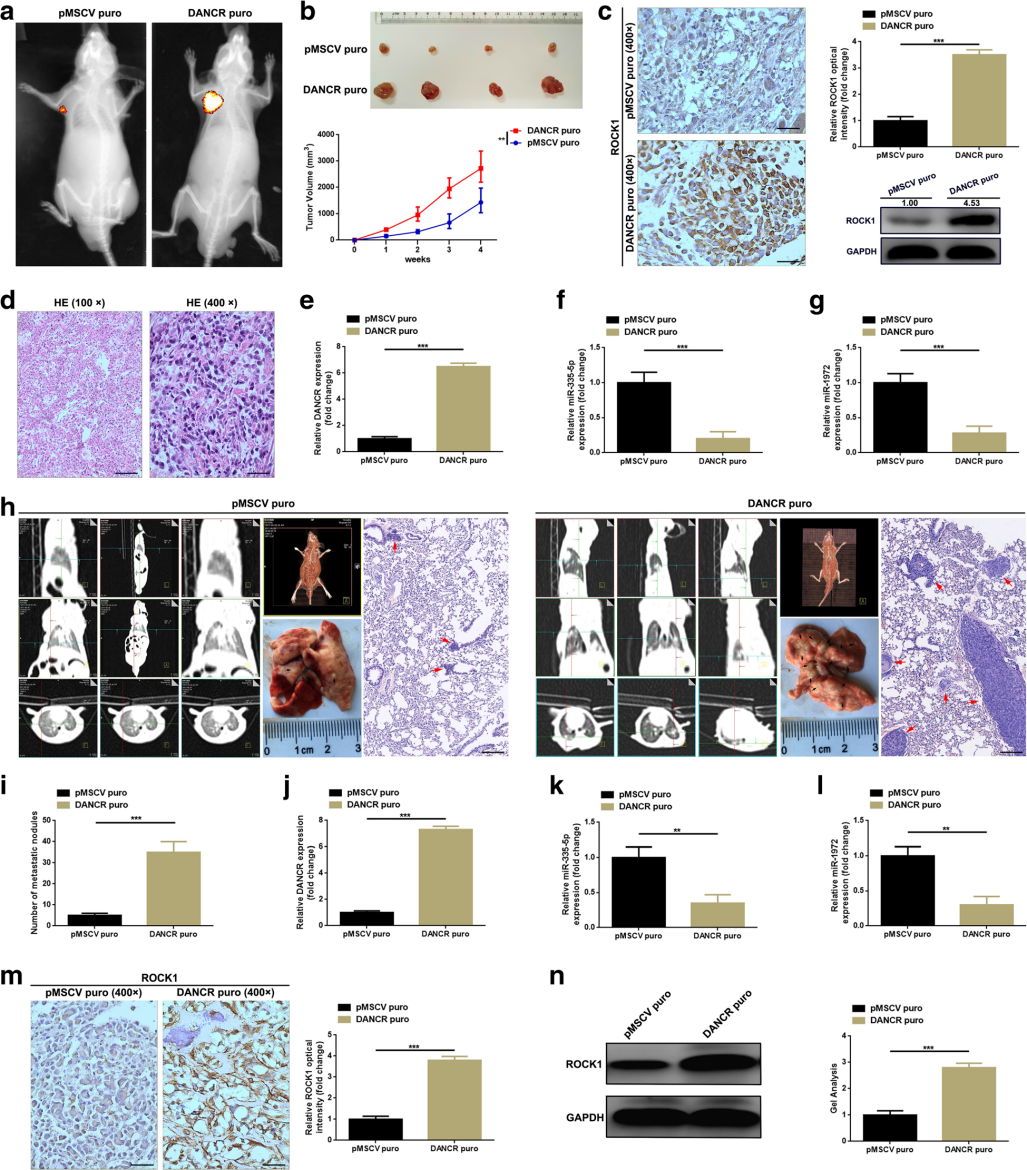
**a**-**b** Representative photographs of IVIS images (**a**), macroscopic appearance (**b** up) and growth curve (**b** down) strongly indicated that elevated DANCR (DANCR puro) promoted tumorigenesis opposite to pMSCV puro group. **c**, **e**-**g** Elevation of DANCR (**e**, confirmed by qRT-PCR) enhanced ROCK1 (**c**, detected by IHC and western blot, scale bars 50 μm) but restricted miR-335-5p (**f**, detected by qRT-PCR) and miR-1972 (**g**, detected by qRT-PCR) expression in the formatted subcutaneous nudes. **d h** & **e** staining demonstrated the formation of osteosarcoma nodules, scale bars, 200 μm and 50 μm for magnifications of 100× and 400×, respectively. **h**-**i** Elevation of DANCR led to promotion of lung metastasis of osteosarcoma which were presented by the representative photographs of CT scan, macroscopic appearance and HE staining (scale bars 200 μm, magnifications of 100×). **j** Elevated expression of DANCR in DANCR puro group was confirmed by qRT-PCR detection compared to pMSCV puro group. **k**-**n** Elevation of DNACR suppressed miR-335-5p (**k**, detected by qRT-PCR) and miR-1972 (**l**, detected by qRT-PCR) but improved ROCK1 (**m** by IHC, scale bars 50 μm, and N by western blot detection) expression in formatted metastatic nudes. All data were normalized to pMSCV puro group, **P < 0.01 as comparing to pMSCV puro group. Data were shown as mean ± SD from three independent experiments

In this section, orthotopic xenograft mouse models were introduced to finally confirm the facilitative effect DANCR conducts on tumor growth and lung metastasis of osteosarcoma. As was shown in Fig. 6a-b, overexpression of DNACR (DANCR puro) significantly promoted tumor growth of ostesoacoma as comparing with blank vector (pMSCV puro) group. Meanwhile, consequence that elevated DANCR but depressed miR-335-5p and miR-1972 in DANCR puro group was noticed by a qRT-PCR detection (Fig. 6e-g). Also, ROCK1 expression in the formatted subcutaneous nudes were determined by IHC and western blot. Data presented in Fig. 6c implied elevation of DNACR could promote ROCK1 expression. Further, at the fourth week after caudal vein injection, the metastatic nudes in lungs were determined by CT scan. As the representative images displayed in Fig. 6h, up-regulating DANCR also contributed to lung metastasis of osteosarcoma in mice. Finally, as the detective results of metastatic nodes in lung demonstrated in Fig. 6i-n, increasing DNACR boosted ROCK1 expression but inhibited miR-335-5p and miR-1972 expression. Together, the indication of this part confirmed that elevation of DANCR promoted tumorigenesis and lung metastasis of osteosarcoma in vivo.

## Discussion

Recently, aberrant expression of some lncRNAs has been elucidated as functioning multiple effects in tumorgenesis of osteosarcoma [22–25]. DANCR is located in human chromosome 4q12, and is reported as an oncogene in diverse carcinomas. Yuan SX suggested that DANCR increased stemness features of hepatocellular carcinoma by derepression of catenin beta 1 (CTNNB1) [26]. Jia J persisted that DANCR promoted the invasion ability of C4-2B and CW22RV1 cells through epigenetically silencing TIMP2/3 expression in a research of prostate cancer [27]. To date, related researches on DNACR and osteosarcoma were rare. Jiang N revealed that DANCR could enhance tumor progression and cancer stemness features in osteosarcoma by upregulating AXL via miR-33a-5p inhibition [28]. In the present study, we took DANCR as a study target based on the previous research foundation. We found that DANCR was raised in osteosarcoma cell lines and in osteosarcoma tissue specimens especially in specimens with late staging, lymph node metastasis and distant metastasis. Also, we validated that elevated DANCR was closely correlated with a short survival time in patients. These data indicated that DANCR works as an oncogene in the progression of osteosarcoma. In the part of clinical detection, a total of 95 patients were involved in our research. The sample size is big enough to illustrate the role of DNACR playing in osteosarcoma. According to the outcomes of clinical detection, the further cellular level of EDU and transwell assays primarily reinforced that DANCR promoted osteosarcoma cells’ proliferation and metastatic abilities. Therefore, exploring out the detailed mechanism of how DANCR working is meaningful.

**Fig. 7:**
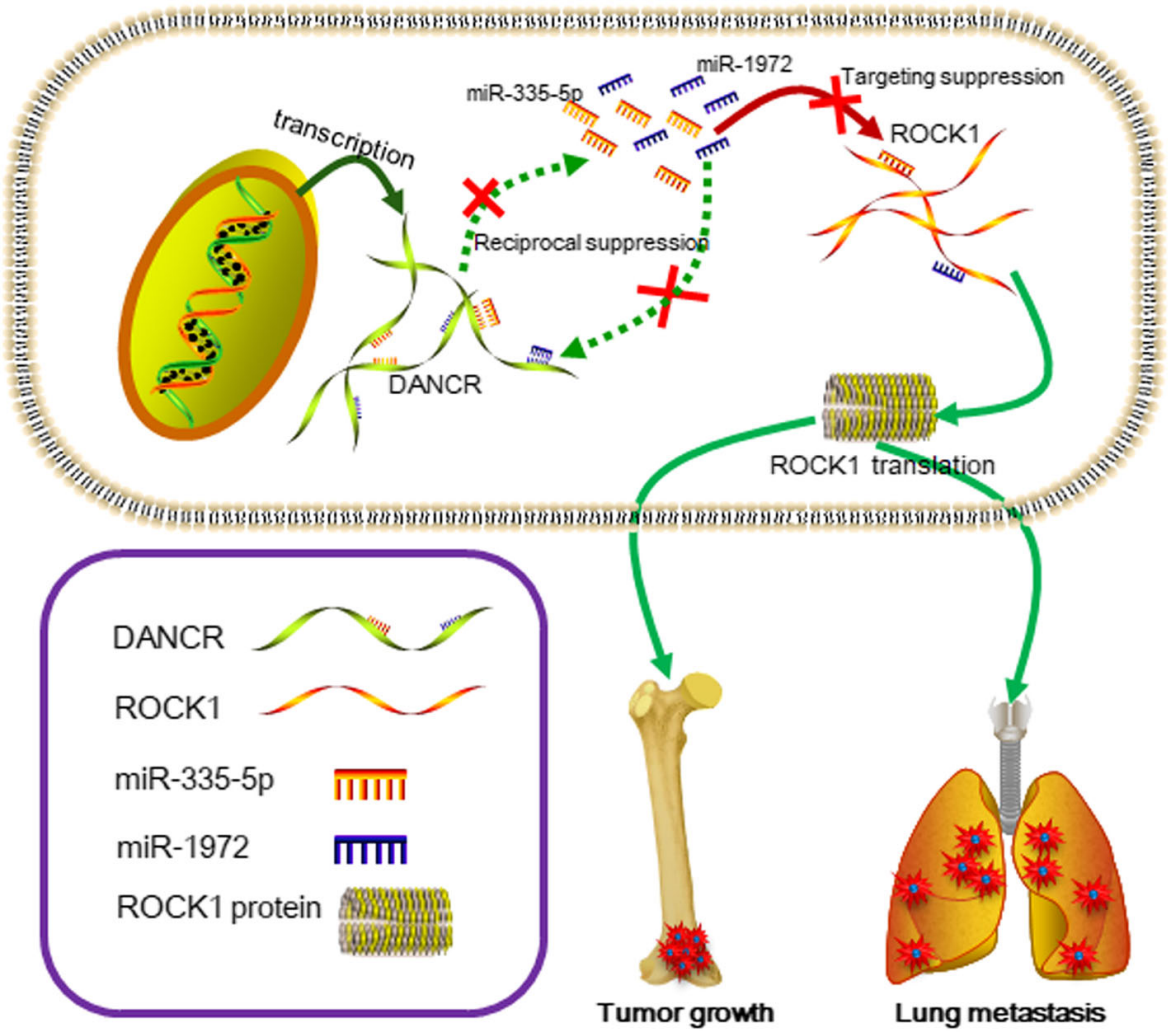
Schematic diagram of mechanism on this research. DANCR promotes ROCK1-mediated proliferation and metastasis via decoying of miR-335-5p and miR-1972 in osteosarcoma

LncRNAs are reported to regulate gene expression and chromatin structure via following ways: decoy effect, scaffold effect and post-transcriptional effect [29]. In 2011, the ceRNA theory was firstly proposed by Leonardo Salmena and was extensively accepted in noncoding RNA area. Numerous researches on ceRNA and cancers were explored [30–36]. In our previous studies, we found that miR-335-5p suppressed osteosarcoma cells’ metastatic ability via targeting its downstream gene ROCK1 [14, 37]. ROCK1 was also broadly reported as a proliferation- and metastasis-related gene in various cancers [38–41]. Hence, we tried to elucidate whether DNACR could affect ROCK1 and its mediated proliferation and metastasis via a same mechanism as ceRNA. As the first encouraging result, we verified that DANCR did promote osteosarcoma cells’ proliferation and metastasis via up-regulation of ROCK1. According to the previous researches, ROCK1 could be targeted by many miRNAs [41–44]. Also, it is well known that lncRNA can regulate numbers of miRNAs at the same time, and one miRNA can regulate an army of downstream target genes. We therefore wondered whether DNACR could co-work with one or more miRNAs to regulate ROCK1-mediated proliferation and metastasis. As a bright result, we found that DANCR and ROCK1 not only shared a similar MREs for miR-335-5p but also shared another MREs for miR-1972. Meanwhile, we revealed the negative correlation between DNACR and miR-335-5p and miR-1972 by a Pearson correlation analysis, respectively. Further, a series of up and down functional experiments clear demonstrated the reciprocal suppressive effect between DNACR and miR-335-5p and miR-1972 respectively which indicating that DANCR could crosstalk with these two miRNAs. It is widely accepted that miRNAs regulated their target genes by directly binding. We here designed a luciferase assay and a RIP binding assay to confirm the targeted binding effect between DANCR/ROCK1 and miR-335-5p/miR-1972 in molecular level, respectively. Convincingly, the constructed luciferase assay and RIP binding assay certified that both DANCR and ROCK1 were the targets of miR-335-5p/miR-1972. In addition, an executed antisense experiment strongly stated that it was wild DANCR cDNA but not mutant DNACR cDNA could reverse the suppressive effect on proliferation and metastasis which was caused by elevation of miR-335-5p or miR-1972. Finally, in an in vivo animal study, we verified again that DNACR positively triggered tumor growth and lung metastasis in mice. Together, as the schematic diagram presented in Fig. 7, we drew the conclusion that DANCR promoted ROCK1-mediated proliferation and metastasis by acting as a ceRNA via decoying of miR-335-5p and miR-1972 in osteosarcoma.

The development and progression of osteosarcoma is a very complicated biological process which including an ocean of molecules and multiple mechanisms. The ceRNA network which are comprised by DNACR, miR-335-5p/miR-1972 and ROCK1 is a hidden corner of iceberg in osteosarcoma. We have reasons to believe that numerous genes and downstream targets of DANCR are still worthy of further exploring.

## Conclusions

The present study firstly demonstrated that DANCR could decoy two miRNAs – miR-335-5p and miR-1972 to facilitate ROCK1-mediated proliferation and metastasis via a ceRNA network. This provide a novel target and a better understanding for possibly potential mechanism of pathogenesis and molecular therapeutic strategy for osteosarcoma.

## Additional files

Additional file 1: **Table S1.** The sequences of primers used in this work. (DOCX 18 kb)

Additional file 2: **Figure S2.** The silenced efficiencies of DANCR and ROCK1 knock down plasmids were qualified by a qRT-PCR assays, and DANCR shRNA-01 as well as ROCK1 shRNA-01 were selected in the following RNAi experiments. (JPG 722 kb)

Additional file 3: **Figure S1.** DIANA TOOLS was applied to predict the potential miRNAs that might interact with DANCR, and the top 18 miRNAs were demonstrated for their higher theoretical binding scores (higher than 0.900). (JPG 3899 kb)

## Abbreviations

3′-UTR: 3′-untranslated region
CeRNA: Competing endogenous RNA
DANCR: Differentiation antagonizing non-protein coding RNA
LncRNAs: Long non-coding RNAs
MiRNAs: microRNAs
MREs: miRNA response elements
RIP assay: RNA immunoprecipitation assay
ROCK1: Rho associated coiled-coil containing protein kinase 1
