# Supplementary material for: Long noncoding RNA DANCR, working as a competitive endogenous RNA, promotes ROCK1-mediated proliferation and metastasis via decoying of miR-335-5p and miR-1972 in osteosarcoma
Source: Mol Cancer. 2018 May 12;17:89. doi: 10.1186/s12943-018-0837-6 (PMC5948795; doi:10.1186/s12943-018-0837-6)
Supplement: Supplementary file 1 — Table S1. The sequences of primers used in this work. (DOCX 18 kb) [file 12943_2018_837_MOESM1_ESM.docx]

| Gene | Sequences of primers used |
| --- | --- |
| DANCR-F | GCGCCACTATGTAGCGGGTT |
| DANCR-R | TCAATGGCTTGTGCCTGTAGTT |
| ROCK1-F | AGGAAGGCGGACATATTAGTCCCT |
| ROCK1-R | AGACGATAGTTGGGTC CCGGC |
| GAPDH-F | GCACCGTCAAGGCTGAGAAC |
| GAPDH-R | TGGTGAAGACGCCAGTGGA |
| miR-335-5p-F | GGGTCAAGAGCAATAACGAA |
| miR-335-5p-R | CAGTGCGTGTCGTGGAGT |
| miR-1972-F | GGGTCAGGCCAGGCACAGT |
| miR-1972-R | CAGTGCGTGTCGTGGAGT |
| U6-F | CTCGCTTCGGCAGCACA |
| U6-R | AACGCTTCACGAATTTGCGT |
| DANCR-01 shRNA | TGAGCCAACTATCCCTTCAGTTACACTTCCTGTCATGTAACTGAAGGGATAGTTGGCTTTTTTTC |
|  | TCGAGAAAAAAAGCCAACTATCCCTTCAGTTACATGACAGGAAGTGTAACTGAAGGGATAGTTGGCTCA |
| DANCR-02 shRNA | TGGCCAAATATGCGTACTAACTTGCTTCCTGTCACAAGTTAGTACGCATATTTGGCCTTTTTTC |
|  | TCGAGAAAAAAGGCCAAATATGCGTACTAACTTGTGACAGGAAGCAAGTTAGTACGCATATTTGGCCA |
| ROCK1-01 shRNA | CCGGCGATTCTATACTGCAGAAGTACTCGAGTACTTCTGCAGTATAGAATCGTTTTTG |
|  | AATTCAAAAACGATTCTATACTGCAGAAGTACTCGAGTACTTCTGCAGTATAGAATCG |
| ROCK1-02 shRNA | CCGGGTTAAGAATCTAACCCTGCAACTCGAGTTGCAGGGTTAGATTCTTAACTTTTTG |
|  | AATTCAAAAAGTTAAGAATCTAACCCTGCAACTCGAGTTGCAGGGTTAGATTCTTAAC |

**Table S1. The sequences of primers used in this work**
