# Supplementary figures and images for: Long noncoding RNA DANCR, working as a competitive endogenous RNA, promotes ROCK1-mediated proliferation and metastasis via decoying of miR-335-5p and miR-1972 in osteosarcoma
Source: Mol Cancer. 2018 May 12;17:89. doi: 10.1186/s12943-018-0837-6 (PMC5948795; doi:10.1186/s12943-018-0837-6)

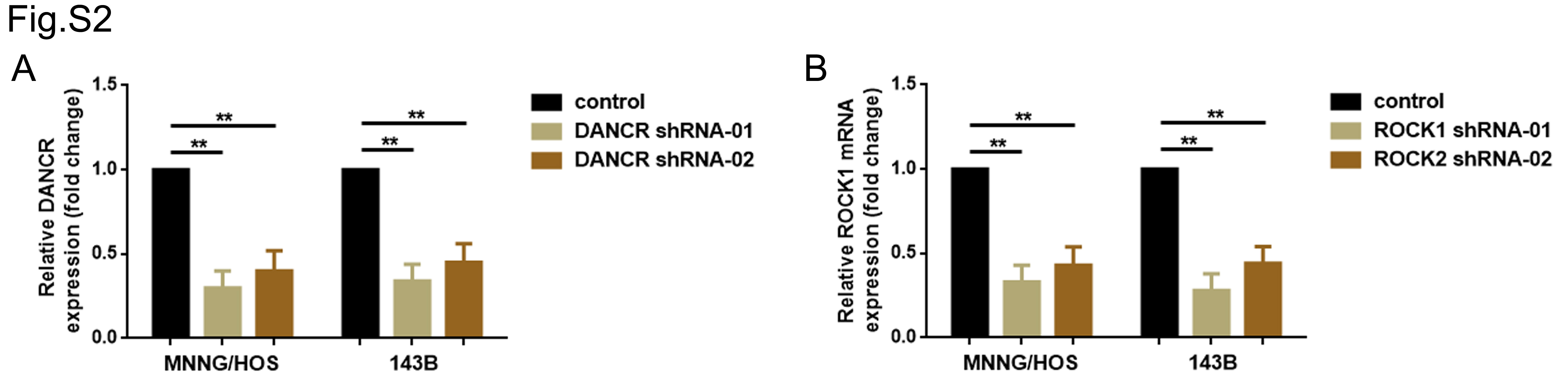

Supplement: Supplementary file 2 — Figure S2. The silenced efficiencies of DANCR and ROCK1 knock down plasmids were qualified by a qRT-PCR assays, and DANCR shRNA-01 as well as ROCK1 shRNA-01 were selected in the following RNAi experiments. (JPG 722 kb) [file 12943_2018_837_MOESM2_ESM.jpg]

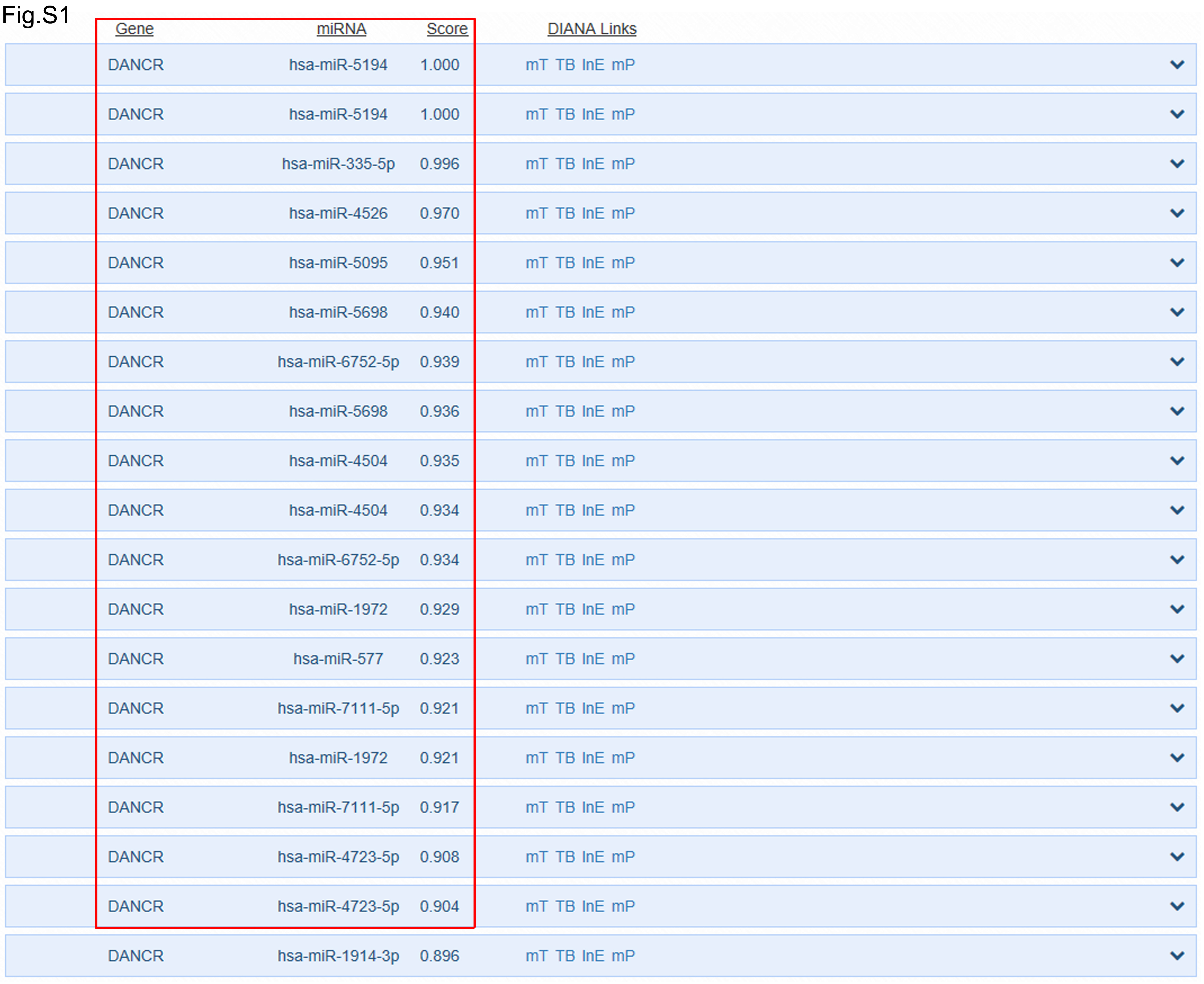

Supplement: Supplementary file 3 — Figure S1. DIANA TOOLS was applied to predict the potential miRNAs that might interact with DANCR, and the top 18 miRNAs were demonstrated for their higher theoretical binding scores (higher than 0.900). (JPG 3899 kb) [file 12943_2018_837_MOESM3_ESM.jpg]
